# The immunomodulation–immunogenicity balance of equine Mesenchymal Stem Cells (MSCs) is differentially affected by the immune cell response depending on inflammatory licensing and major histocompatibility complex (MHC) compatibility

**DOI:** 10.3389/fvets.2022.957153

**Published:** 2022-10-20

**Authors:** Alina Cequier, Francisco José Vázquez, Antonio Romero, Arantza Vitoria, Elvira Bernad, Mirta García-Martínez, Isabel Gascón, Laura Barrachina, Clementina Rodellar

**Affiliations:** ^1^Laboratorio de Genética Bioquímica LAGENBIO, Instituto de Investigación Sanitaria de Aragón (IIS), Instituto Agroalimentario de Aragón-IA2 (Universidad de Zaragoza-CITA), Zaragoza, Spain; ^2^Servicio de Cirugía y Medicina Equina, Hospital Veterinario, Universidad de Zaragoza, Zaragoza, Spain

**Keywords:** mesenchymal stem cells, horse, allogeneic therapy, haplotype, co-culture, immune response, gene expression, mediator secretion

## Abstract

The immunomodulatory properties of equine mesenchymal stem cells (MSCs) are important for their therapeutic potential and for their facilitating role in their escape from immune recognition, which may also be influenced by donor–recipient major histocompatibility complex (MHC) matching/mismatching and MHC expression level. Factors such as inflammation can modify the balance between regulatory and immunogenic profiles of equine MSCs, but little is known about how the exposure to the immune system can affect these properties in equine MSCs. In this study, we analyzed the gene expression and secretion of molecules related to the immunomodulation and immunogenicity of equine MSCs, either non-manipulated (MSC-naive) or stimulated by pro-inflammatory cytokines (MSC-primed), before and after their exposure to autologous or allogeneic MHC-matched/-mismatched lymphocytes, either activated or resting. Cytokine priming induced the immunomodulatory profile of MSCs at the baseline (MSCs cultured alone), and the exposure to activated lymphocytes further increased the expression of *interleukin 6 (IL6), cyclooxygenase 2*, and *inducible nitric oxide synthase*, and IL6 secretion. Activated lymphocytes were also able to upregulate the regulatory profile of MSC-naive to levels comparable to cytokine priming. On the contrary, resting lymphocytes did not upregulate the immunomodulatory profile of equine MSCs, but interestingly, MSC-primed exposed to MHC-mismatched lymphocytes showed the highest expression and secretion of these mediators, which may be potentially linked to the activation of lymphocytes upon recognition of foreign MHC molecules. Cytokine priming alone did not upregulate the immunogenic genes, but MSC-primed exposed to activated or resting lymphocytes increased their *MHC-I* and *MHC-II* expression, regardless of the MHC-compatibility. The upregulation of immunogenic markers including *CD40* in the MHC-mismatched co-culture might have activated lymphocytes, which, at the same time, could have promoted the immune regulatory profile aforementioned. In conclusion, activated lymphocytes are able to induce the equine MSC regulatory profile, and their effects seem to be additive to the priming action. Importantly, our results suggest that the lymphocyte response against MHC-mismatched MSC-primed would promote further activation of their immunomodulatory ability, which eventually might help them evade this reaction. Further studies are needed to clarify how these findings might have clinical implications *in vivo*, which will help developing safer and more effective therapies.

## Introduction

Mesenchymal stem cells (MSCs) are of great interest to treat several pathologies, including musculoskeletal injuries such as those affecting the horse, which is a species of remarkable value as both patient and translational models (1, 2). The regulatory and immunomodulatory properties of MSCs are currently considered their main therapeutic mechanism and involve both direct cell-to-cell contact and contact-independent paracrine signaling, *via* the expression of adhesion molecules like vascular cell adhesion molecule 1 (*VCAM1*) and the secretion of molecules such as interleukin 6 (IL6) and prostaglandin E2 (PGE_2_) (3), respectively (4). Since the immunomodulatory properties of equine MSCs might have profound therapeutic implications in the treatment of many inflammatory-mediated processes in the horse, a growing number of studies have focused on analyzing such immune properties (4). To elucidate possible pathways for immunosuppression exerted by equine MSCs, and how these are influenced by different factors, it is critical to study the expression and secretion of mediators implied in their paracrine mechanisms, including the enzymes producing these molecules, such as cyclooxygenase 2 (*COX2*), indoleamine 2,3-dioxygenase 1 (*IDO1*), or inducible nitric oxide synthase 2 (*iNOS2*).

Furthermore, the MSC immunomodulatory activity is not only important for its therapeutic mechanism but also for its facilitating role in its escape from immune recognition when administered allogenically. Allogeneic application presents several advantages over autologous therapy as it increases the availability of thoroughly characterized cells for therapy, particularly when autologous cells are not suitable because of genetic or metabolic diseases, or in aged patients (5). However, MSCs are no longer considered truly immune-privileged but are considered immune-evasive, so their recognition and elimination by the immune system after their allogeneic administration should be considered (6). Allogeneic MSCs may be rejected due to the expression of foreign antigens on their surface, which may raise both cellular and humoral immune responses against the cells (7) and even lead to immune memory mechanisms that could prevent effective and safe repeated administration of allogeneic MSCs in the horse (8).

The surface expression of major histocompatibility complex (MHC) class I and II antigens on equine MSCs facilitates their immune recognition by lymphocytes, and antibodies can be generated specifically directed against the equine leukocyte antigen (ELA) of the donor, potentially compromising the therapeutic effectiveness of the cells. Therefore, MHC matching between the donor and recipient is receiving increasing attention in the last few years, and ELA haplotypes have been taken into account in equine studies (9, 10), as well as in other species (11, 12). It should be noted that the MHC haplotype is a factor intrinsic to each individual and as such cannot be modified. Furthermore, it has also been reported that the MHC level expression in MSCs in basal conditions is quite dependent on the equine donor (9). The knowledge on these factors is critical to design better therapeutic strategies, including donor selection. As a matter of fact, some researchers are exploring a possible link between low MHC antigen expression and universal blood types to select equine donors whose MSCs would defer immune recognition (7).

Nevertheless, there are other factors that may modify the inherent immune properties of MSCs, such as their exposure to an inflammatory environment. Priming MSCs with pro-inflammatory cytokines like interferon gamma (IFNγ) and tumor necrosis factor alpha (TNFα) could increase their immunomodulatory properties and may result in enhanced regulatory effects *in vivo* (13). However, priming MSCs may also raise their immunogenicity by inducing the expression of MHCs, thus potentially limiting their allogeneic administration (14). These changes in MSC immune properties upon inflammatory exposure are influenced by the type and duration of priming. For example, while priming with high concentration of IFNγ can increase *MHC-II* gene expression in equine MSCs (9), priming with low doses of IFNγ and TNFα for a short period upregulated several immune regulatory-related genes without significantly increasing the expression of immunogenic markers (15). However, while a significant advance has been made on how different cytokines and ligands may influence the immune properties of equine MSCs, the effects of an immune response environment on MSCs have been less explored.

To develop allogeneic cell therapies is critical to gain knowledge of how factors such as MHC matching/mismatching and inflammation may affect the balance between the immunomodulatory and immunogenic potentials of equine MSCs. Such immune properties can be assessed by evaluating the proliferation of lymphocytes exposed to MSCs in immunosuppression assays or in modified one-way mixed lymphocyte reactions (MLRs) (5). Our group recently reported the effects of equine MSCs on different lymphocyte subpopulations after their *in vitro* co-culture with autologous or allogeneic MHC-matched/-mismatched MSCs, either unstimulated or primed with pro-inflammatory cytokines (16). These *in vitro* assays provide important information on the changes experienced by lymphocytes after contacting with MSCs, contributing to understand the immune response *in vivo*. However, little is known about how MSCs behave when they are exposed to lymphocytes, either if these are already activated during a disease or if they become activated in response to MSCs.

To better understand the fate of MSCs when they enter into contact with the immune system, this study aimed at analyzing the changes in the gene expression and secretion of molecules related to the immune regulatory and immunogenic profiles of equine MSCs after being exposed to activated or resting lymphocytes. Our specific goals were to evaluate the influence of inflammation and compatibility for the MHC in different *in vitro* co-culture settings. For these purposes, equine MSCs in basal conditions (MSC-naive) or pro-inflammatory primed (MSC-primed) were co-cultured with autologous or allogeneic MHC-matched/-mismatched lymphocytes in both immunosuppression assays (activated lymphocytes) and in modified one-way MLRs (resting lymphocytes). Subsequently, MSC gene expression and secretion of different molecules related to their immunomodulatory–immunogenicity balance were assessed.

Our initial hypothesis was that exposure of equine MSCs to lymphocytes would result in increased gene expression and secretion of immunomodulatory molecules accompanied by a slight upregulation of their immunogenic profile. We hypothesized that these changes would be more marked in MSC-primed, particularly after contacting with activated lymphocytes. Regarding the MHC compatibility, it was hypothesized that MSCs exposed to MHC-matched lymphocytes would display a profile similar to that in the autologous setting, whereas the MHC-mismatched co-cultures would result in similar immunomodulation but increased immunogenicity of the equine MSCs.

## Materials and methods

### Study design

**Figure 1:**
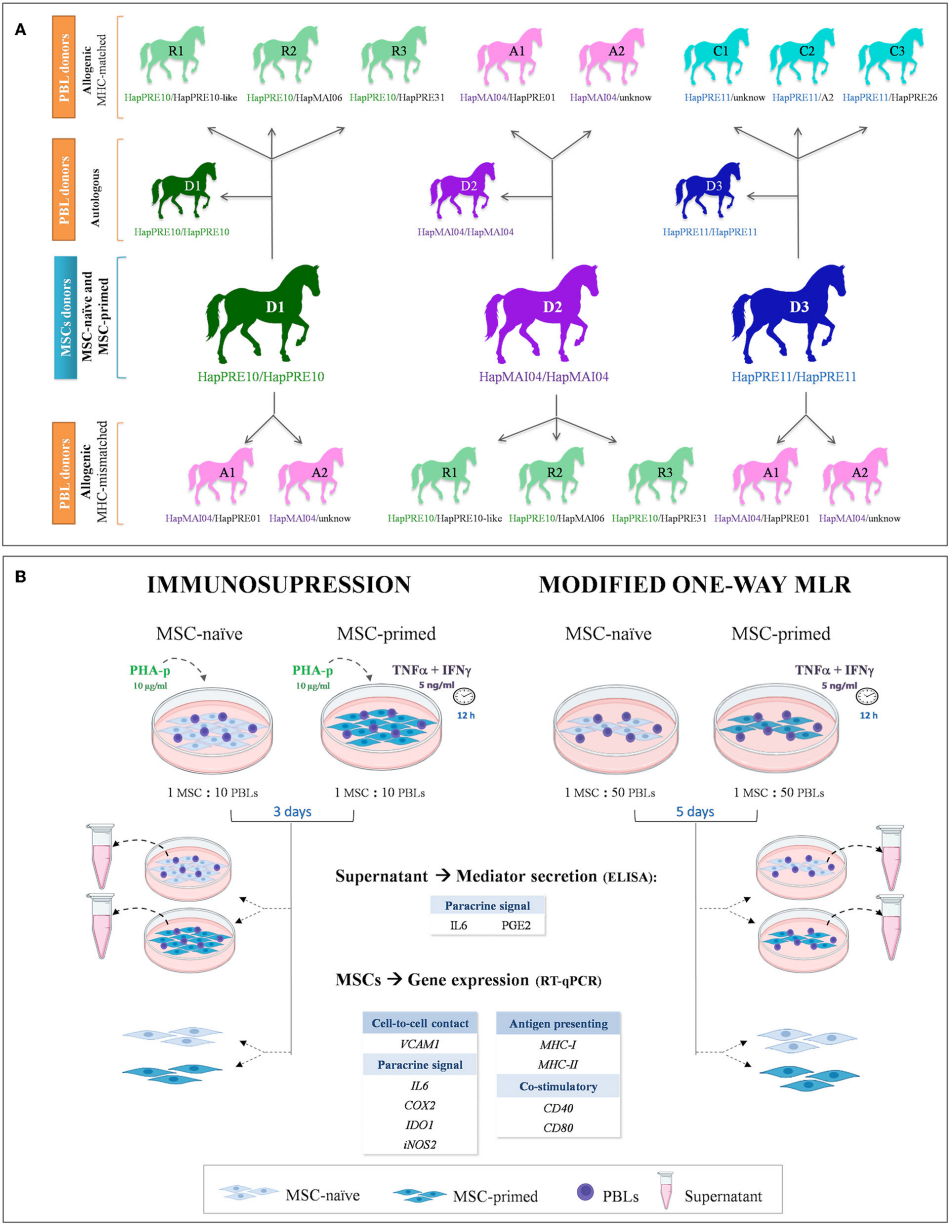
Study design. **(A)** Equine donors of mesenchymal stem cells (MSCs) and peripheral blood lymphocytes (PBLs) are presented with their ELA haplotypes and the combinations to establish autologous and allogeneic major histocompatibility complex (MHC)-matched and MHC-mismatched co-cultures. **(B)** Equine MSCs were assayed with unstimulated (MSC-naive) and primed with cytokines (MSC-primed) in both immunosuppression and modified one-way mixed lymphocyte reaction (MLR) assays, using PBLs activated by phytohemagglutinin isoform P (PHA-p) or resting lymphocytes, respectively. After co-culture, supernatant and MSCs were collected to evaluate, respectively, the secretion and gene expression of different mediators involved in the immunomodulatory capacity and immunogenic potential of these cells.

Equine bone marrow (BM)-derived MSCs were obtained from three MHC homozygous donors and were assayed in both basal conditions (MSC-naive) and after pro-inflammatory priming (MSC-primed). MSC-naive and MSC-primed from each donor were co-cultured with peripheral blood lymphocytes (PBLs), either autologous (*n* = 3) or allogeneic from MHC-matched (*n* = 8) and -mismatched (*n* = 7) animals. These PBLs were obtained from the three MSC donors (autologous setting) and from eight horses selected by their MHC haplotype to establish different allogeneic matched and mismatched combinations, as shown in Figure 1A. A total of two types of co-cultures were used: immunosuppressive assays, where PBLs were previously activated, and modified one-way MLR, where resting PBLs were used. After each type of co-culture, PBLs were removed and used in a separate study (16), MSCs were harvested to analyze their gene expression, and supernatants collected to assess their secretion. The gene expression of different molecules related to the immunomodulatory (*VCAM1, COX2, IDO1, iNOS2, IL6*) and immunogenic (*MHC-I, MHC-II, CD40, CD80*) profiles of MSCs were evaluated by real-time quantitative polymerase chain reaction (RT-qPCR), and secretion of IL6 and PGE_2_ was determined by ELISA in the supernatants (Figure 1B).

### Animal selection by MHC haplotyping

In total, 11 mixed-breed horses (one stallion, three geldings, seven mares; aged 2–8 years, weight 412–493 kg) in good health status and with no previous pregnancy history were chosen based on their MHC haplotypes. To find and select animals, a screening of 60 Purebred Spanish and mixed-breed horses from a local farm was performed. Haplotypes were determined by microsatellite typing using a validated panel of 10 highly polymorphic intra-MHC regions, as previously described (8, 17). Blood was collected after owner's informed consent, and methodology for DNA extraction, multiplex PCRs, and fragment analysis was performed, as previously reported by our group (8).

Definitive haplotypes were established for homozygous animals, and the remaining animals were assigned with provisional haplotypes based on previously known ones, which are either reported in the literature (10, 18) or described in a preliminary study of our group in Purebred Spanish horses (19). Overall, three groups of animals were selected, with each group including one homozygous horse of the haplotype HapPRE10, HapPRE11, or HapMAI04, and two or three heterozygous animals sharing one haplotype with the donor. Thus, the homozygous horse in each group served as the MSC donor as it was MHC-matched with the heterozygous animals. To establish MHC-mismatched combinations, PBLs from the heterozygous animals in other groups with different haplotypes were used (Figure 1A). Selecting homozygous individuals as MSC donors allows matching them with different heterozygous individuals. This strategy has been proposed to create haplo-banks of human-induced pluripotent stem cells (iPSCs) (20, 21) and has also been used in equine MSC studies (22). Supplementary Table 1 shows the microsatellite alleles of each haplotype identified in the horses involved in this study.

All procedures involving animals were carried out under the Project License PI 15/16 approved by the in-house Advisory Ethics Committee for Animal Research from the University of Zaragoza. The care and use of animals were performed in according with the Spanish Policy for Animal Protection RD53/2013, which is in line with the European Union Directive 2010/63 on the protection of animals used for scientific purposes. All animals were kept on paddocks of the facilities of the Animal Research Service of the University of Zaragoza, with free access to water and grass hay.

#### Isolation, characterization, and culture of MSCs

Equine bone marrow MSCs were obtained and characterized, as previously described (15) as part of a previous study of our group (16). In brief, bone marrow was harvested from the sternum of D1, D2, and D3 animals under sedation (0.04 mg/kg IV romifidine, Sedivet, Boehringer Ingelheim, and 0.02 mg/kg IV butorphanol, Torbugesic, Pfizer) and local analgesia with lidocaine (Anesvet, Laboratorios Ovejero). Mononuclear cells were separated by density gradient centrifugation and seeded in the culture medium consisting of low-glucose Dulbecco's modified Eagle medium supplemented with 2 mM L-glutamine, 0.1 mg/mL streptomycin, 100 U/mL penicillin, and 10% fetal bovine serum (FBS) (all from Sigma-Aldrich). The cells were expanded until passage three and characterized by their phenotype and tri-lineage differentiation. Characterization data of the MSC lines used in this study were previously published by our group (16). Subsequently, the MSCs were cryopreserved in 10% dimethyl sulfoxide (DMSO) (Sigma-Aldrich) and 90% FBS medium until the experiments started.

Prior to co-cultures, the cryopreserved MSCs (*n* = 3) were thawed and seeded at 5,000 cells/cm^2^ in the basal medium, as described above, at 37°C and 5% CO_2_ for 72 h to recover from freezing. At 24 h prior to co-culture, the MSCs were detached with 0.25% trypsin–EDTA (Sigma–Aldrich) and seeded into a 24-well plate at 100,000 cells per well for the immunosuppression assays, and at 20,000 MSCs per well for modified one-way MLR assays.

For the MSC-primed condition, the basal media described above was supplemented with 5 ng/mL of TNFa (R&D Systems) plus 5 ng/mL of IFNg (R&D Systems) and corresponding MSCs were exposed for 12 h to this media, as described earlier (15), before adding PBLs.

#### Blood collection and isolation of PBLs

Peripheral blood lymphocytes were isolated using the carbonyl iron granulocyte depletion method, followed by density gradient centrifugation with Lymphoprep^TM^, as previously described (8, 23). In brief, blood was collected aseptically *via* a jugular venipuncture into sterile 60-mL syringes with 17 IU/mL of lithium heparin (Sigma-Aldrich), and plasma was allowed to separate for 20 min at room temperature (RT). Plasma was separately collected into conical tubes using extension sets and incubated with carbonyl iron (Sigma-Aldrich) in agitation for 30 min at 37°C. Then, carbonyl iron was placed at the bottom of the tubes by using a magnet, and supernatant was collected and centrifuged at 310 × g for 5 min. The cellular pellet was resuspended in PBS and overlayed on Lymphoprep^TM^. After centrifugation at 690 × g for 15 min (without brake), the lymphocyte layer was recovered and washed with PBS. The cells were counted in a hemocytometer chamber using 0.4% trypan blue as dye exclusion. This isolation technique has been reported to provide an enriched lymphocyte population (95–99%) (8, 24).

### Co-cultures of MSCs with lymphocytes: Immunosuppression assays and modified one-way MLR

#### Co-culture of MSCs with activated lymphocytes: Immunosuppression assay

To simulate the environment of an immune response, MSCs were exposed to activated lymphocytes by conducting immunosuppression assays. As described before, corresponding MSCs were previously plated in a 24-well plate at 100,000 cells per well in duplicate and prepared for each condition (MSC-naive and MSC-primed). Lymphocytes from autologous, MHC-matched and -mismatched horses were seeded at 1 × 10^6^ PBLs per well (1:10 ratio MSC:PBL), based on previous studies (25, 26) according to the combinations presented in Figure 1. The PBL medium used for co-culture consisted of RPMI 1640 (Thermo Fisher) supplemented with 10% FBS, 0.1 mM 2-mercaptoethanol, 100 U/mL penicillin, and 100 μg/mL streptomycin (all from Sigma-Aldrich). The PBL medium was supplemented with 10 μg/mL of the mitogen phytohemagglutinin isoform P (PHA, Sigma-Aldrich) (27, 28) to activate lymphocyte proliferation.

MSC-naive and MSC-primed were cultured alone in the same conditions to provide baseline measurements for both gene expression and molecule secretion. Appropriate controls were set along with experimental conditions in duplicate. Lymphocytes from all animals, either PHA-activated or unstimulated, were cultured alone as positive and negative controls, respectively, to account for their possible contribution to the secretion of molecules measured by ELISA. All the experimental co-cultures and controls were maintained for 3 days, after which corresponding analyses were performed, as will be detailed in the following text.

#### Modified one-way MLR

Modified one-way MLRs were performed by co-culturing MSCs with resting (unstimulated) lymphocytes. This setting aims at reflecting what would happen to MSCs if these are recognized by the immune system and raise a response that can simultaneously change the MSC profile. Stimulator MSCs, either naive or primed, were previously plated at 20,000 cells per well on 24-well plates in duplicate for each condition, as described previously. Autologous, MHC-matched and -mismatched responder PBLs were seeded at 1 × 10^6^ PBL per well according to the combinations depicted in Figure 1, thus resulting in an MSC/PBL ratio of 1:50 (9, 26).

Positive and negative controls were set by establishing, respectively, matched and mismatched classic MLRs using responder PBLs from each donor. In brief, MHC-matched or -mismatched PBLs were used as stimulators by treating them with 50 μg/mL mitomycin C (Sigma-Aldrich) (37°C 30 min incubation, followed by two washes with PBS at 310 × g 5 min) to inhibit proliferation (10, 29). Stimulator PBLs and responder PBLs were cultured at a ratio of 1:1. The supernatant from the MHC-matched and -mismatched MLRs was used to account for potential contribution of lymphocytes to the secretion of the analyzed molecules. All the co-cultures and controls were maintained for 5 days without media exchange, and corresponding analyses were performed subsequently, as detailed in the following text.

### Analysis of expression of genes involved in equine immune response (RT-qPCR)

The expression level of genes coding for immunosuppression- and immunogenicity-related molecules was evaluated in MSC-naive and MSC-primed cultured alone (baseline) and after being co-cultured with autologous, MHC-matched or -mismatched PBLs in both immunosuppression and modified one-way MLR. After the co-cultures, PBLs were removed, and MSCs were washed with PBS and frozen at −80°C until mRNA was extracted. MSCs cultured alone for baseline were processed in the same way.

Isolation of mRNA and complementary DNA (cDNA) synthesis were performed using the Cells-to-cDNA II kit (Ambion) according to the manufacturer's instructions, and RT-qPCRs were performed and monitored with a QuantStudio 3 Real-Time PCR System (Applied Biosystems). All reactions were carried out in a total volume of 10 μL with 2 μL of cDNA as the template and Fast SYBR Green Master Mix (Applied Biosystems). Amplification was performed in triplicate for each sample as follows: 20 s at 95°C for initial activation, followed by 40 cycles consisting of 3 s at 95°C and 30 s at 60°C, and a dissociation curve protocol run after every PCR.

The levels of gene expression were determined by using the comparative ΔΔCt method. As a reference sample, values from MSC-naive cultured alone (baseline) from each donor were used, unless otherwise stated. The normalization factor was calculated as the geometric mean of the quantity of two housekeeping genes, glyceraldehyde 3-phosphate dehydrogenase (*GAPDH*) and beta-2-microglobulin (*B2M*). Genes were analyzed, and corresponding primer sequences were previously designed by our group (15) and are presented in Table 1, grouped according to their function.

**Table 1:** Primers used for gene expression analysis by RT-qPCR.

**Gene**	**Accession number**	**Primer sequence (5^′^–3^′^)**	**Amplicon size (bp)**
**Housekeeping**			
*GAPDH*	NM_001163856	F: GGCAAGTTCCATGGCACAGT R: CACAACATATTCAGCACCAGCAT	128
*B2M*	NM_001082502	F: TCGTCCTGCTCGGGCTACT R: ATTCTCTGCTGGGTGACGTGA	102
**Immunomodulation-related molecules**			
***Molecules related with cell-to-cell contact mechanism***			
*VCAM1*	NM_001101650	F: TCTATGCTACGCTCTGGCTACG R: TTGATGGTCTCCCCGATGA	127
***Molecules related with paracrine signaling mechanism***			
COX2	AB041771	F: GTTTGCATTTTTTGCCCAGC R: ACTTAAATCCACCCCGTGACC	103
*IDO1*	XM_014736538.2	F: TCATGACTACGTGGACCCAAAA R: CGCCTTCATAGAGCAGACCTTC	104
*iNOS2*	AY027883	F: CCAACAATGGCAACATCAGGT R: TGAGCATTCCAGATCCGGA	85
*IL6*	EU438770	F: AACAGCAAGGAGGTACTGGCA R: CAGGTCTCCTGATTGAACCCA	95
**Immunogenic markers:** ***Antigen presenting-related molecules***			
*MHC-I*	AB525081	F: CGTGAGCATCATTGTTGGC R: TCCCTCTTTTTTCACCTGAGG	92
*MHC-II*	NM_001142816	F: AGCGGCGAGTTGAACCTACAGT R: CGGATCAGACCTGTGGAGATGA	172
**Antigen-presenting-related molecules: *Co-Stimulatory molecules***			
*CD40*	AY514017	F: ACAAATACTGCGACCCCAACC R: TTTCACAGGCATCGCTGGA	114
*CD80*	XM_005601958.3	F: CAGGAAAGTTGGCTCTGACCA R: TCTCCATTGTGATCCTGGCTC	135

GenBank accession numbers of the sequences used for primers design. Primers (F: forward and R: reverse) and length of the amplicon in base pair (bp). Genes were grouped in agreement with the functions and implications of encoded molecules. *GAPDH*, glyceraldehyde 3-phosphate dehydrogenase; *B2M*, beta-2-microglobulin; *VCAM1*, vascular cell adhesion molecule 1; *COX2*, cyclooxygenase 2; *IDO1*, indoleamine 2 3-dioxygenase 1; *iNOS2*, inducible nitric oxide synthase 2; *IL6*, interleukin 6; *MHC-I*, major complex of histocompatibility I; *MHC-II*, major complex of histocompatibility II; *CD40*, cluster of differentiation 40; *CD80*, cluster of differentiation 80.

### Assessment of interleukin 6 and prostaglandin E2 secretion

Supernatants collected from MSC-PBL co-cultures were used to evaluate PGE_2_ and IL6 production by using commercially available ELISA kits, as previously reported (26, 29–31). The secretion of these molecules was assessed at the baseline (MSC-naive and MSC-primed cultured alone) and after exposure to the different types of PBLs in both immunosuppression and modified one-way MLR assays. The supernatants from unstimulated and PHA-stimulated PBLs seeded alone were used as negative and positive controls, respectively, for the immunosuppression assays. For the modified one-way MLR assays, the supernatants from the classical MLRs with MHC-matched or -mismatched PBLs as stimulators were used as negative and positive controls, respectively.

After all co-cultures, PBLs were collected, centrifuged at 310 × g for 5 min, and the supernatants recovered. Supernatants were recovered in the same way from controls consisting of PBLs alone and from classic MLRs, as well as from MSC-naive and MSC-primed cultured alone. All the supernatants were centrifuged at 500 × g for 15 min to remove any contaminating cell and subsequently frozen at −20°C for further ELISA. All the procedures were performed as per the manufacturer's instructions and concentrations determined using a standard curve, including a blank.

For PGE_2_ analysis (Prostaglandin E2 Parameter Assay Kit, R&D Systems, Ref: KGE004B), control supernatants were diluted 1:10, baseline supernatants were diluted 1:2, and supernatants from co-cultures were diluted 1:50 in the reagent diluent. The standard curve was established from 39 to 5,000 pg/mL. For IL6 analysis (Equine IL-6 DuoSet ELISA, R&D Systems, REF: DY1886), baseline and control supernatants were diluted 1:1 in the reagent diluent, and supernatants from co-cultures were not diluted. The standard curve was set from 62.5 to 16,000 pg/mL. All the samples and points of the standard curve were run in duplicate. All the colorimetric assays were analyzed on a microplate reader SPECTROstar Nano (BMG LABTECH) and read immediately at 450 nm with wavelength correction set to 540 nm. The duplicate readings for each standard, control, and sample were averaged, and the average zero standard optical density was extracted. The standard curve was created generating a four-parameter logistic curve fit, and the concentrations extrapolated were multiplied by the dilution factor. Samples with values beyond the limit of detection were excluded from the analysis.

### Statistical analysis

Statistical analysis was performed by GraphPad Prism 9.2 software (San Diego, CA, USA). Results of RT-qPCR and ELISA were separately analyzed for each type of co-culture and not directly compared as the experimental conditions were different. Analytical statistics were performed to check differences in mRNA relative expression or molecule secretion depending on the different study variables. The independent variables were “group” (three categories: autologous, allogenic MHC-matched, and allogenic MHC-mismatched co-cultures) and “cell type” (two categories: MSC-naive and MSC-primed). The existence of outlier samples was evaluated with the Grubbs test (alpha = 0.05). For comparisons between three or more groups, normality and homoscedasticity were evaluated by using the Shapiro–Wilk test and the Levene test, respectively. When data followed a normal distribution and had homogeneous variances, the parametric test ANOVA was used, followed by Tukey's comparisons test as a *post-hoc*. In normally distributed data with unequal variances, Welch's *t*-test was used. In non-normal data, the non-parametric Kruskal–Wallis test was used, followed by Dunn's test as a *post hoc*. The effect of the type of co-culture was analyzed by comparing the results for each type of combination (autologous, MHC-matched, and MHC-mismatched) for each type of MSC (MSC-naive and MSC-primed) using parametric or non-parametric paired tests. The significance level was set at *p* < 0.05 for all analyses.

## Results

The expression level of different genes and the secretion of molecules were evaluated in MSCs from three donors under different conditions: MSC-naive and MSC-primed cultured alone (baseline) and after being co-cultured with autologous, and MHC-matched or mismatched PHA-activated PBLs (immunosuppression assays) or resting PBLs (modified one-way MLR assays). The expression level of five genes involved in the immunomodulatory properties of equine MSCs was analyzed (*IL6, COX2, IDO1, iNOS2*, and *VCAM1)*, along with the secretion of IL6 and PGE_2_. Also, to account for the effect of the different conditions on MSC immunogenicity, four genes coding for antigen-presenting-related molecules were assessed: *MHC-I, MHC-II, CD80*, and *CD40*.

For gene expression, data are presented as relative expression (fold change) over corresponding baseline MSC-naive, unless otherwise stated. Baseline values are the same for both types of assays (immunosuppression and modified one-way MLR). For molecule secretion, the supernatants from lymphocytes cultured alone in resembling conditions were used as controls. The IL6 and PGE_2_ concentration detected in the lymphocyte controls for the immunosuppression assay (unstimulated, CTL–; PHA-activated, CTL+) and for the modified one-way MLR assays (classic MLRs) was very low and significantly different from that measured in the co-culture supernatants, confirming that MSCs were the major contributors to IL6 and PGE_2_ secretion in the co-cultures. The details on these significant differences can be found in Supplementary Figure 1. Note that the scale in Y axes of the graphs presenting results of gene expression and molecule secretion in immunosuppression and modified one-way MLR assays is different to better show the values.

### Gene expression and secretion of mediators related to equine MSC immunomodulation

**Figure 2:**
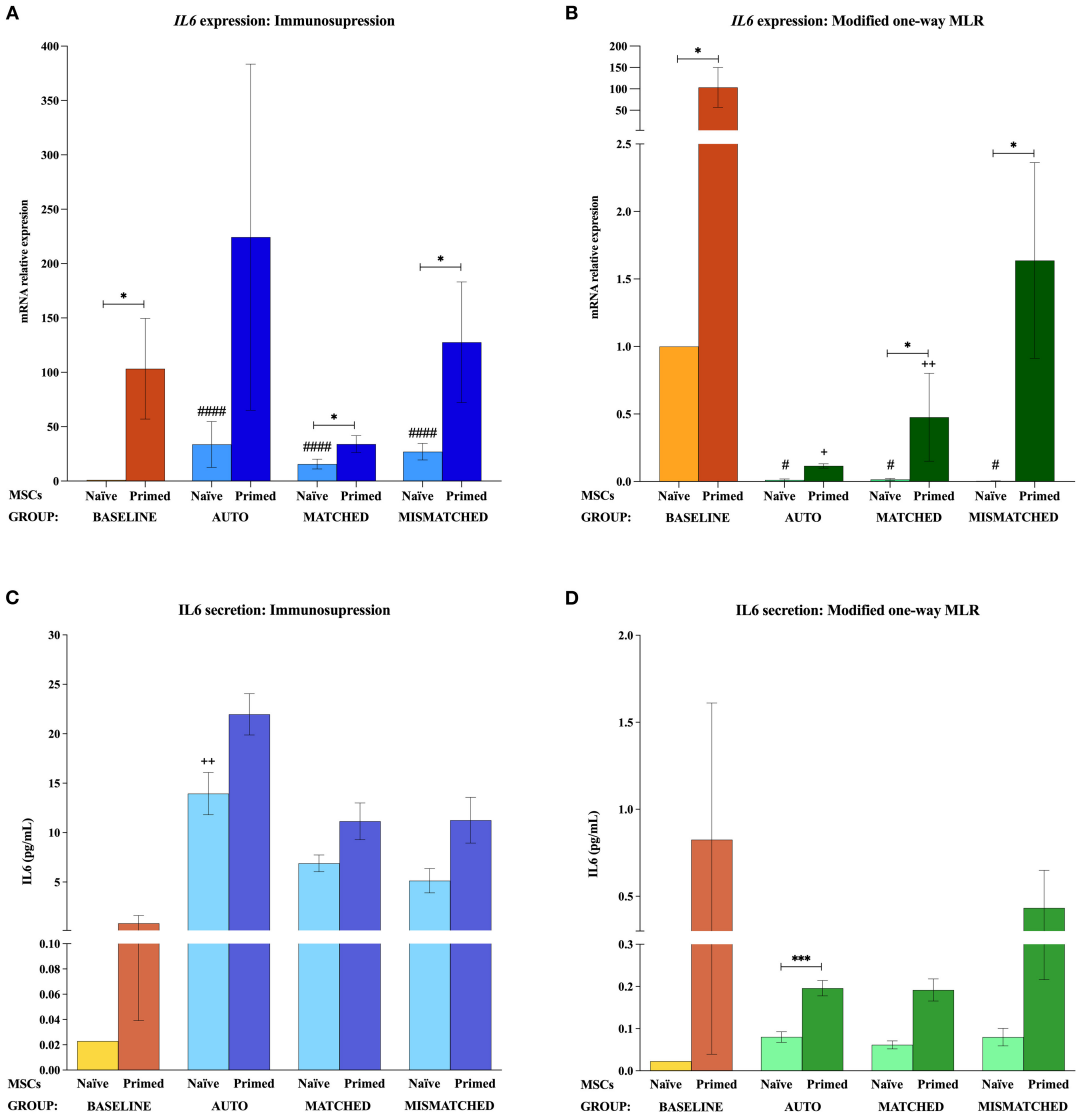
Interleukin 6 (IL6) gene expression and secretion by equine mesenchymal stem cells (MSCs) in the different scenarios. **(A)** *IL6* mRNA relative expression and **(C)** IL6 secretion (pg/mL) before (baseline; orange bars) and after equine MSC-naive (light blue bars) and MSC-primed (dark blue bars) were exposed *in vitro* to phytohemagglutinin (PHA)-activated peripheral blood lymphocytes (PBLs) (immunosuppression assays). **(B)** *IL6* mRNA relative expression and **(D)** IL6 secretion before (baseline; orange bars) and after MSC-naive (light green bars) and MSC-primed (dark green bars) were exposed *in vitro* to resting PBLs [modified one-way mixed lymphocyte reaction (MLR) assays]. Co-cultures of MSCs and PBLs were autologous (*n* = 3), allogeneic, matched (*n* = 8), or mismatched (*n* = 7) for the major histocompatibility complex. Changes in gene expression are represented as mean ± S.E.M of the relative mRNA expression, using baseline MSC-naive as reference sample (light orange bar, value 1). Concentration of IL6 in the supernatant from the different conditions is represented as mean ± S.E.M (pg/mL). Significant differences of each condition compared with the baseline MSC-naive (light orange bar) are represented by hashes (#) above the corresponding bar (^#^*p* < 0.05; ^*####*^*p* < 0.0001). Significant differences compared with the baseline MSC-primed (dark orange bar) are represented by a cross (+) above the corresponding bar (^+^*p* < 0.05; ^++^*p* < 0.01). Significant differences between experimental conditions are represented by a squared line with an asterisk (^*^p < 0.05; ^***^p < 0.001).

**Figure 3:**
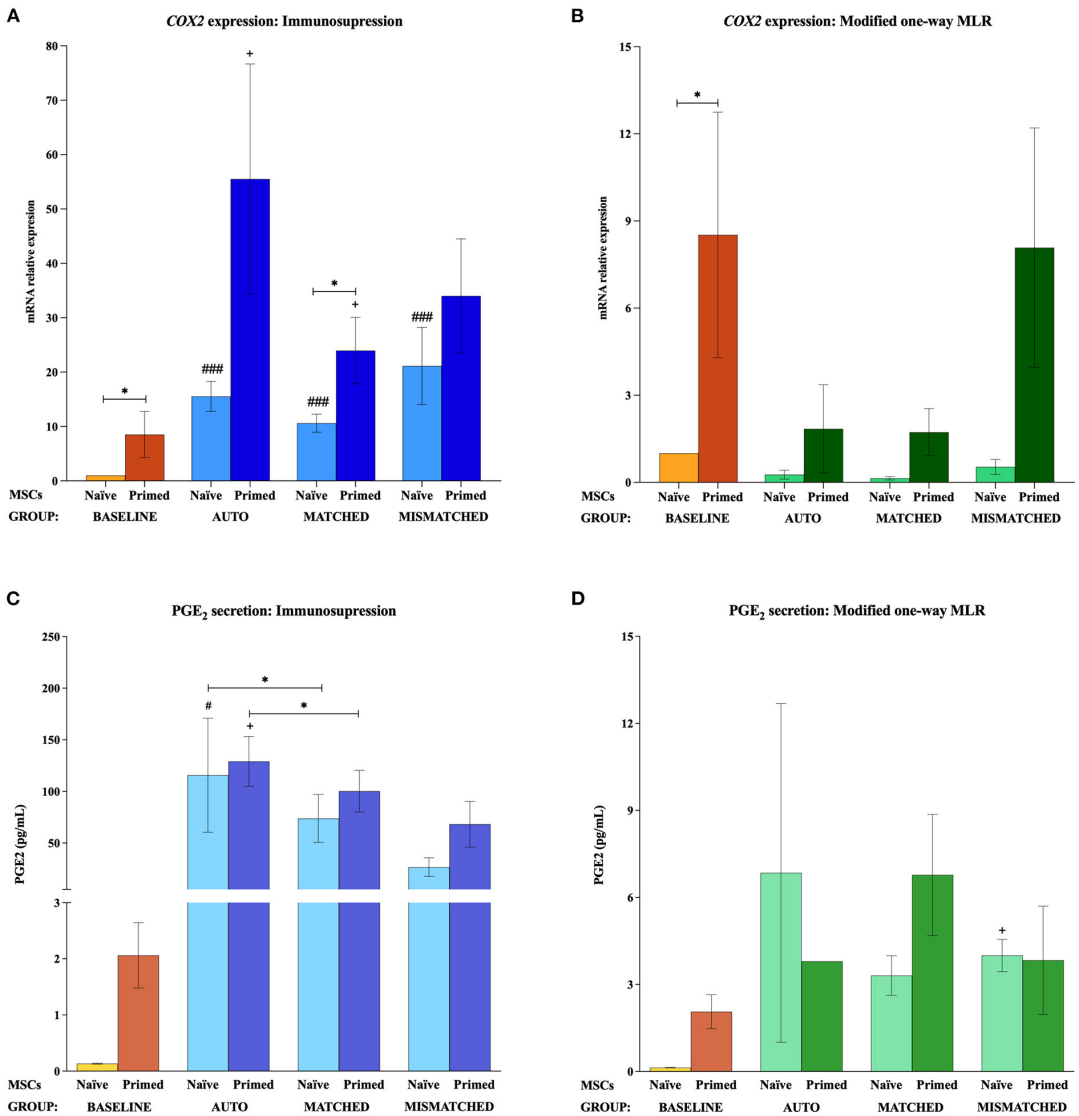
*Cyclooxygenase 2 (COX2)* gene expression and prostaglandin E2 (PGE_2_) secretion by equine mesenchymal stem cells (MSCs) in the different scenarios. **(A)** *COX2* mRNA relative expression and **(C)** PGE_2_ secretion (pg/mL) before (baseline; orange bars) and after equine MSC-naive (light blue bar) and MSC-primed (dark blue bar) were exposed *in vitro* to phytohemagglutinin (PHA)-activated peripheral blood lymphocytes (PBLs) (immunosuppression assays). **(B)** *COX2* mRNA relative expression and **(D)** PGE_2_ secretion before (baseline; orange bars) and after MSC-naive (light green bar) and MSC-primed (dark green bar) were exposed *in vitro* to resting PBLs [modified one-way mixed lymphocyte reaction (MLR) assays]. Co-cultures of MSCs and PBLs were autologous (*n* = 3) or allogeneic, matched (*n* = 8), or mismatched (*n* = 7) for the major histocompatibility complex. Changes in gene expression are represented as mean ± S.E.M of the relative mRNA expression, using baseline MSC-naive as reference sample (light orange bar, value 1). Concentration of PGE_2_ in the supernatant from the different conditions is represented as mean ± S.E.M (pg/mL). Significant differences of each condition compared with the baseline MSC-naive (light orange bar) are represented by hashes (#) above the corresponding bar (^#^*p* < 0.05; ^*###*^*p* < 0.001). Significant differences compared with the baseline MSC-primed (dark orange bar) are represented by a cross (+) above the corresponding bar (^+^*p* < 0.05). Significant differences between experimental conditions are represented by a squared line with an asterisk (^*^p < 0.05).

**Figure 4:**
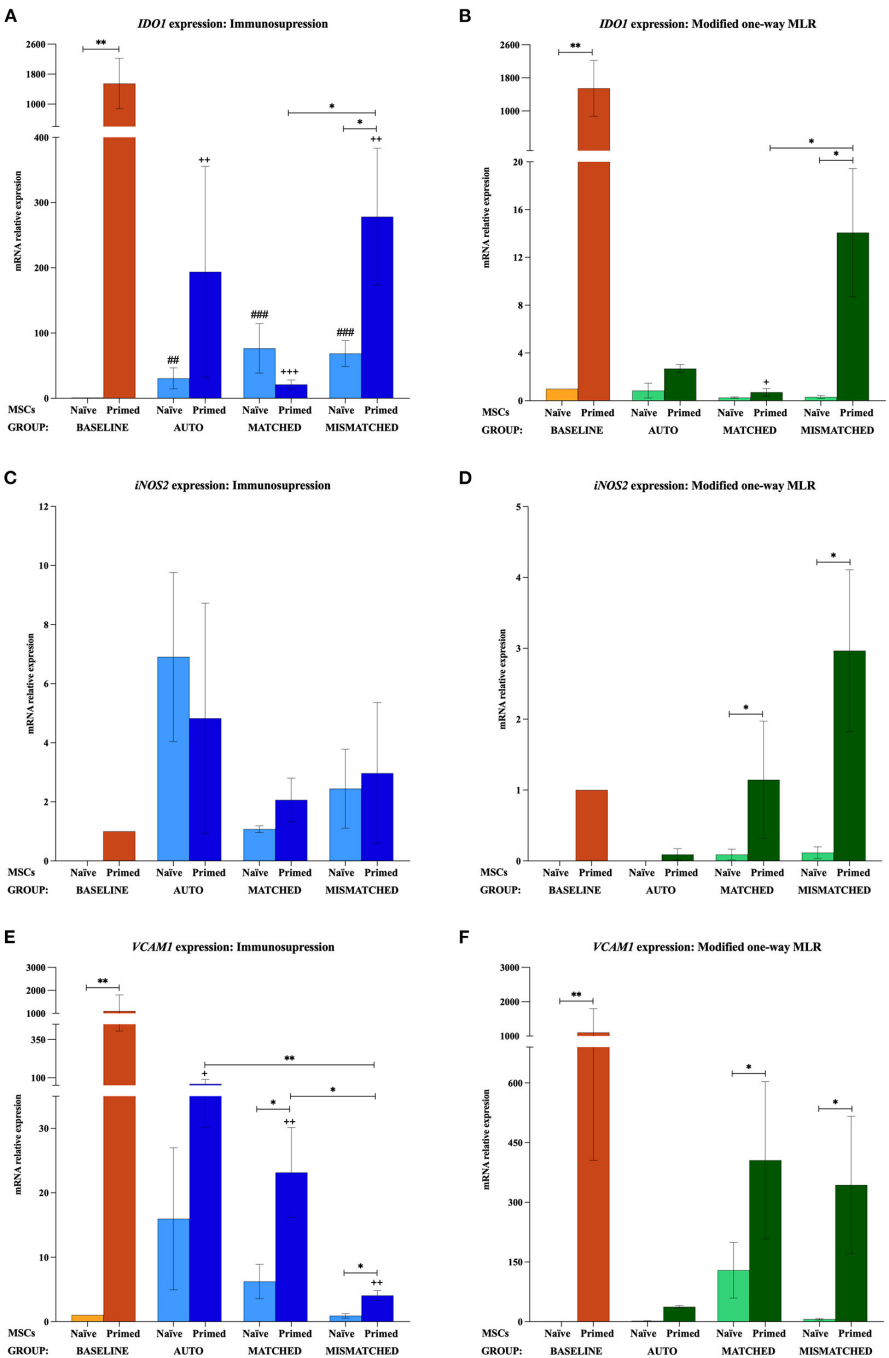
Changes in indoleamine 2,3-dioxygenase 1 (*IDO1*), inducible nitric oxide synthase 2 (*iNOS2*), and vascular cell adhesion molecule 1 (*VCAM1*) expression by equine mesenchymal stem cells (MSCs) in the different scenarios **(A)** *IDO1*, **(C)** *iNOS2*, and **(E)** *VCAM1* mRNA relative expression before (baseline; orange bars) and after equine MSC-naive (light blue bars) and MSC-primed (dark blue bars) were exposed *in vitro* to phytohemagglutinin (PHA)-activated peripheral blood lymphocytes (PBLs) (immunosuppression assays). **(B)** *IDO1*, **(D)** *iNOS2*, and **(F)** *VCAM1* mRNA relative expression before (baseline; orange bars) and after MSC-naive (light green bars) and MSC-primed (dark green bars) were exposed *in vitro* to resting PBLs [modified one-way mixed lymphocyte reaction (MLR) assays]. Co-cultures of MSCs and PBLs were autologous (*n* = 3) or allogeneic, matched (*n* = 8), or mismatched (*n* = 7) for the major histocompatibility complex. Changes in *IDO1* and *VCAM1* expression are represented as mean ± S.E.M of the relative mRNA expression, using baseline MSC-naive as reference sample (light orange bar, value 1). Baseline MSC-primed are used as reference sample (dark orange bar, value 1) for *iNOS2* since no expression of this gene was detected in baseline MSC-naive. Significant differences of each condition compared with the baseline MSC-naive (light orange bar) are represented by hashes (#) above the corresponding bar (^*##*^*p* < 0.01; ^*###*^*p* < 0.001). Significant differences compared with the baseline MSC-primed (dark orange bar) are represented by a cross (+) above the corresponding bar (^+^*p* < 0.05; ^++^*p* < 0.01; ^+++^*p* < 0.001). Significant differences between experimental conditions are represented by a squared line with an asterisk (^*^p < 0.05; ^**^p < 0.01).

At the baseline (MSCs cultured alone), cytokine priming induced a significant upregulation of all the immunomodulatory genes studied: *IL6* (*p* < 0.05; Figures 2A,B), *COX2* (*p* < 0.05; Figures 3A,B), *IDO1* (*p* < 0.01; Figures 4A,B), and *VCAM1* (*p* < 0.01; Figures 4E,F). The expression of *iNOS2* was not detected in MSC-naive at the baseline, but it was activated in MSC-primed. Therefore, the expression of *iNOS2* is presented as relative expression (fold change) over baseline MSC-primed (cultured alone), instead of MSC-naive. Following the same trend, the stimulation with cytokines induced IL6 and PGE_2_ secretion in MSC-primed at the baseline, but significant differences could not be found over MSC-naive as these molecules were only detected in MSC-naive from one donor and at very low concentration (Figures 2C,D, 3C,D).

#### Effect of activated lymphocytes on the equine MSC immunomodulatory profile

The exposure of MSC-naive to activated PBLs upregulated the expression of different immunomodulatory genes compared with the baseline, regardless of the compatibility scenario (autologous or allogeneic MHC-matched/-mismatched). Specifically, MSC-naive exposed to activated lymphocytes significantly upregulated *IL6* (*p* < 0.0001 in all conditions; Figure 2A), *COX2* (*p* < 0.001 in all conditions; Figure 3A), and *IDO1* (autologous, *p* < 0.01; matched, *p* < 0.001; mismatched, *p* < 0.001; Figure 4A). *iNOS* and *VCAM1* expression also increased compared with the baseline, but these changes were not statistically significant (Figures 4C,E).

MSC-primed exposed to activated lymphocytes showed higher expression than MSC-naive for *IL-6* (matched and mismatched co-cultures, *p* < 0.05; Figure 2A) and *COX2* (matched co-culture, *p* < 0.05; Figure 3A). However, compared with the baseline (MSC-primed alone), the levels of *IL-6* were not further increased by the presence of activated lymphocytes (Figure 2A). On the other hand, *COX2* did experience a significant upregulation compared with the baseline in autologous and MHC-matched co-cultures (*p* < 0.05 for both conditions; Figure 3A). *iNOS2* was also overexpressed after exposure of MSC-primed to activated lymphocytes, but the differences compared with the baseline were not statistically significant (Figure 4C). In contrast to *IL6, COX2*, and *iNOS2, IDO1* and *VCAM1* were markedly downregulated in MSC-primed exposed to all the types of activated lymphocytes: autologous (*IDO1, p* < 0.01; *VCAM1, p* < 0.05), matched (*IDO1, p* < 0.001; *VCAM1, p* < 0.01), and mismatched (*IDO1, p* < 0.01; *VCAM1, p* < 0.01; Figures 4A,E). Interestingly, there was a clear trend for *VCAM1* downregulation depending on the type of activated lymphocytes, with the mismatched co-culture leading to the greatest reduction (*p* < 0.05 compared with matched, *p* < 0.01 compared with autologous; Figure 4E). Nevertheless, both *IDO1* and *VCAM1* remained higher in MSC-primed than in MSC-naive. This difference was significant for *IDO1* in the mismatched co-cultures (*p* < 0.05), which was also higher than that for the matched co-cultures (*p* < 0.05; Figure 4A) and for *VCAM1* in both allogeneic matched and mismatched co-cultures (*p* < 0.05 for both conditions; Figure 4E). Finally, differences in the expression of *IL6* and *COX2* were not found between MSCs exposed to autologous, matched and mismatched co-cultures, neither for MSC-naive nor for MSC-primed (Figures 2A, 3A).

In agreement with gene expression changes, the secretion of IL6 and PGE_2_ increased compared with the baseline when MSCs were exposed to activated lymphocytes, suggesting that this environment activates MSC immunomodulatory potential. MSC-primed tended to produce more IL-6 and PGE_2_ than MSC-naive, but significant differences were not observed as in gene expression. The highest concentrations of these molecules were found in the autologous co-cultures, which produced significantly more IL6 (MSC-naive, *p* < 0.01; Figure 2C) and PGE_2_ (MSC-naive and MSC-primed, *p* < 0.05) than the baseline and the matched co-culture (PGE_2_, MSC-naive and MSC-primed, *p* < 0.05; Figure 3C).

#### Effect of resting lymphocytes on equine MSC immunomodulatory profile

In contrast to that observed after the exposure to activated lymphocytes, equine MSCs co-cultured with resting lymphocytes showed a downregulation of their immunomodulatory profile. MSC-naive notably downregulated the expression of *IL6* (*p* < 0.05 in all conditions; Figure 2B) and *COX2* (non-significant; Figure 3B), while *IDO1* expression remained low in all the three types of co-cultures (Figure 4B). The expression of *iNOS2* was not detected in MSC-naive exposed to autologous resting lymphocytes and was low in the matched and mismatched co-cultures (Figure 4D). On the contrary, *VCAM1* was upregulated in MSC-naive after being in contact with resting lymphocytes, but these changes were not statistically significant (Figure 4F). In terms of secretion, IL6 was not significantly induced in MSC-naive by resting lymphocytes, and only a slightly higher amount of this molecule was detected after the co-cultures (Figure 2D). In contrast to that observed for *COX2* gene expression, the exposure of MSC-naïve to resting lymphocytes led to an increased PGE_2_ secretion compared with the baseline, although, in general, at lower levels than their exposure to activated lymphocytes. Specifically, this increase was statistically significant when MSC-naïve were exposed to resting mismatched lymphocytes (*p* < 0.05; Figure 3D).

Overall, the expression of immunomodulatory genes remained higher in MSC-primed than in MSC-naïve in all the co-cultures with resting lymphocytes (*IL-6*, matched and mismatched, *p* < 0.05, Figure 2B; *COX2*, non-significant, Figure 3B; *IDO1*, mismatched, *p* < 0.05, Figure 4B; *iNOS2*, matched and mismatched, *p* < 0.05, Figure 4D; *VCAM1*, matched and mismatched, *p* < 0.05, Figure 4F). Nevertheless, and similarly to MSC-naive, MSC-primed co-cultured with autologous and matched resting lymphocytes showed a reduced expression of *IL6* (*p* < 0.05 and *p* < 0.01, respectively; Figure 2B), *COX2* (non-significant; Figure 3B), and *IDO1* (matched co-culture, *p* < 0.05; Figure 4B). Accordingly, co-cultured MSC-primed also decreased their secretion of IL6 compared with the baseline but still secreted higher concentrations of this molecule than MSC-naïve (*p* < 0.001 in autologous settings; Figure 2D). Interestingly however, MSC-primed co-cultured with mismatched resting lymphocytes showed the highest expression of *IL6, COX2, IDO1*, and *iNOS2* among all the co-cultures, even though the differences were not statistically significant, except for *IDO1* (compared with matched co-culture, *p* < 0.05; Figure 4B). Furthermore, the same was observed in terms of IL6 secretion, with the highest concentration of this molecule being produced by MSC-primed exposed to mismatched lymphocytes (Figure 2D), although the difference was not statistically significant. Even though this was also the case for *COX2* gene expression, the same was not replicated at the level of PGE_2_ secretion, and the production of this molecule did not follow a clear trend (Figure 3D).

### Gene expression of markers related to equine MSC immunogenicity

The analysis of *MHC-I* could only be carried out with the MSCs from two of the donors (D2 and D3) since the MSC-naive and MSC-primed from the other donor (D1) did not express *MHC-I* neither at the baseline (MSCs cultured alone) nor after their exposure to autologous or allogeneic MHC-matched lymphocytes, regardless of these being activated or resting. Interestingly, *MHC-I* gene expression was detected in the MSCs of this donor after these were co-cultured with allogeneic MHC-mismatched lymphocytes, either activated or resting. However, the lack of reference values to establish the relative expression of *MHC-I* prevented to include the data from the D1 donor in the analysis. Even though data from only two donors were used for *MHC-I*, a consistent trend on its expression could be observed, which was very similar to that for *MHC-II* and *CD40*.

**Figure 5:**
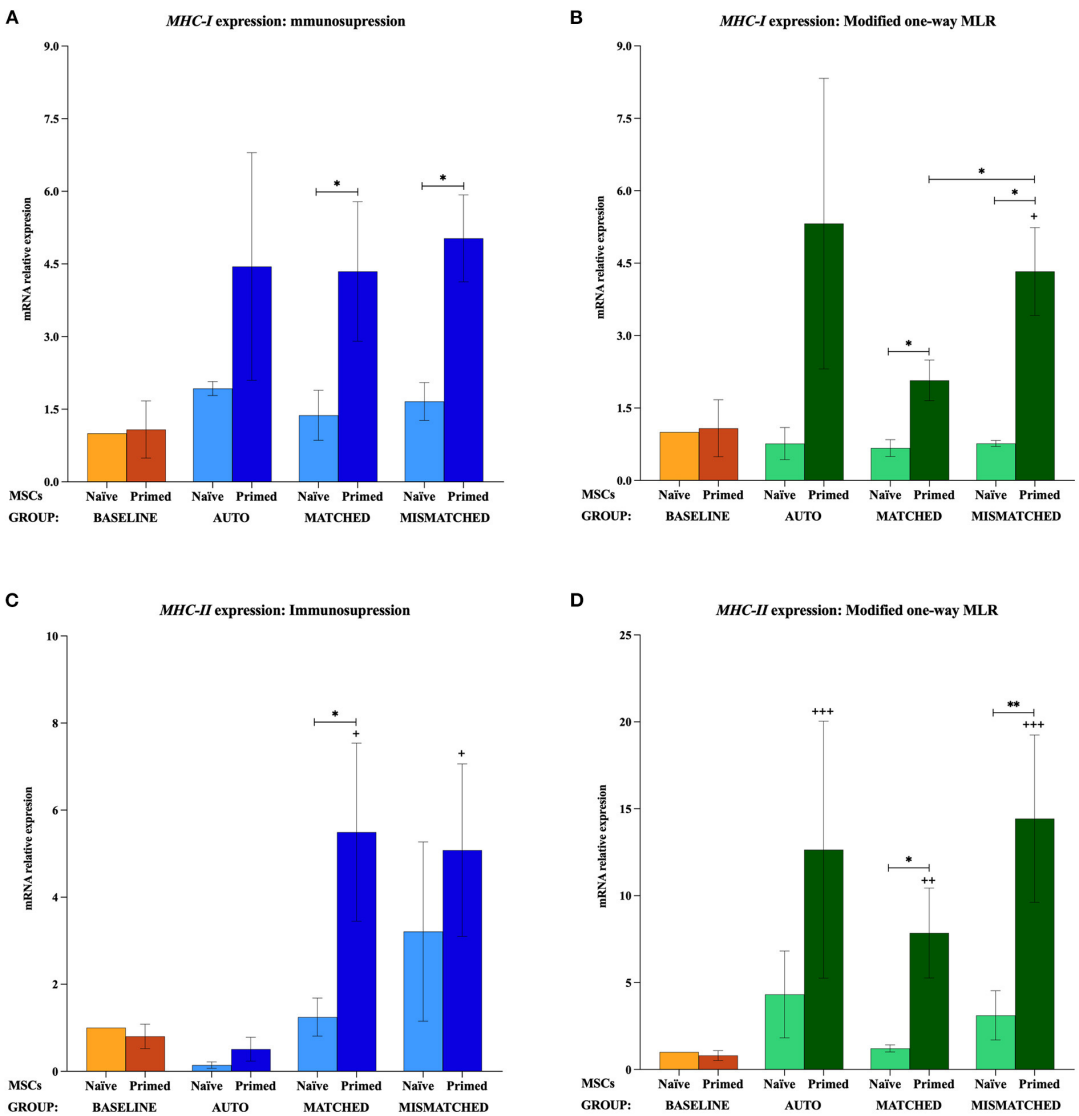
Changes in major histocompatibility complex (*MHC*)-*I* and MHC-*II* expression by equine mesenchymal stem cells (MSCs) in the different scenarios. **(A)** *MHC-I* and **(C)** *MHC-II* mRNA relative expression before (baseline; orange bars) and after equine MSC-naive (light blue bars) and MSC-primed (dark blue bars) were exposed *in vitro* to phytohemagglutinin (PHA)-activated peripheral blood lymphocytes (PBLs) (immunosuppression assays). **(B)** *MHC-I* and **(D)** *MHC-II* mRNA relative expression before (baseline; orange bars) and after MSC-naive (light green bars) and MSC-primed (dark green bars) were exposed *in vitro* to resting PBLs [modified one-way mixed lymphocyte reaction (MLR) assays]. Co-cultures of MSCs and PBLs were autologous (*n* = 3) or allogeneic, matched (*n* = 8), or mismatched (*n* = 7) for the MHC. Data from the D1 donor could not be included in the *MHC-I* analysis because of the lack of reference values since this gene was not expressed at the baseline. Changes in gene expression are represented as mean ± S.E.M of the relative mRNA expression, using baseline MSC-naive as reference sample (light orange bar, value 1). Significant differences compared with the baseline MSC-primed (dark orange bar) are represented by a cross (+) above the corresponding bar (^+^*p* < 0.05; ^++^*p* < 0.01; ^+++^*p* < 0.001). Significant differences between experimental conditions are represented by a squared line with an asterisk (^*^p < 0.05; ^**^p < 0.01).

**Figure 6:**
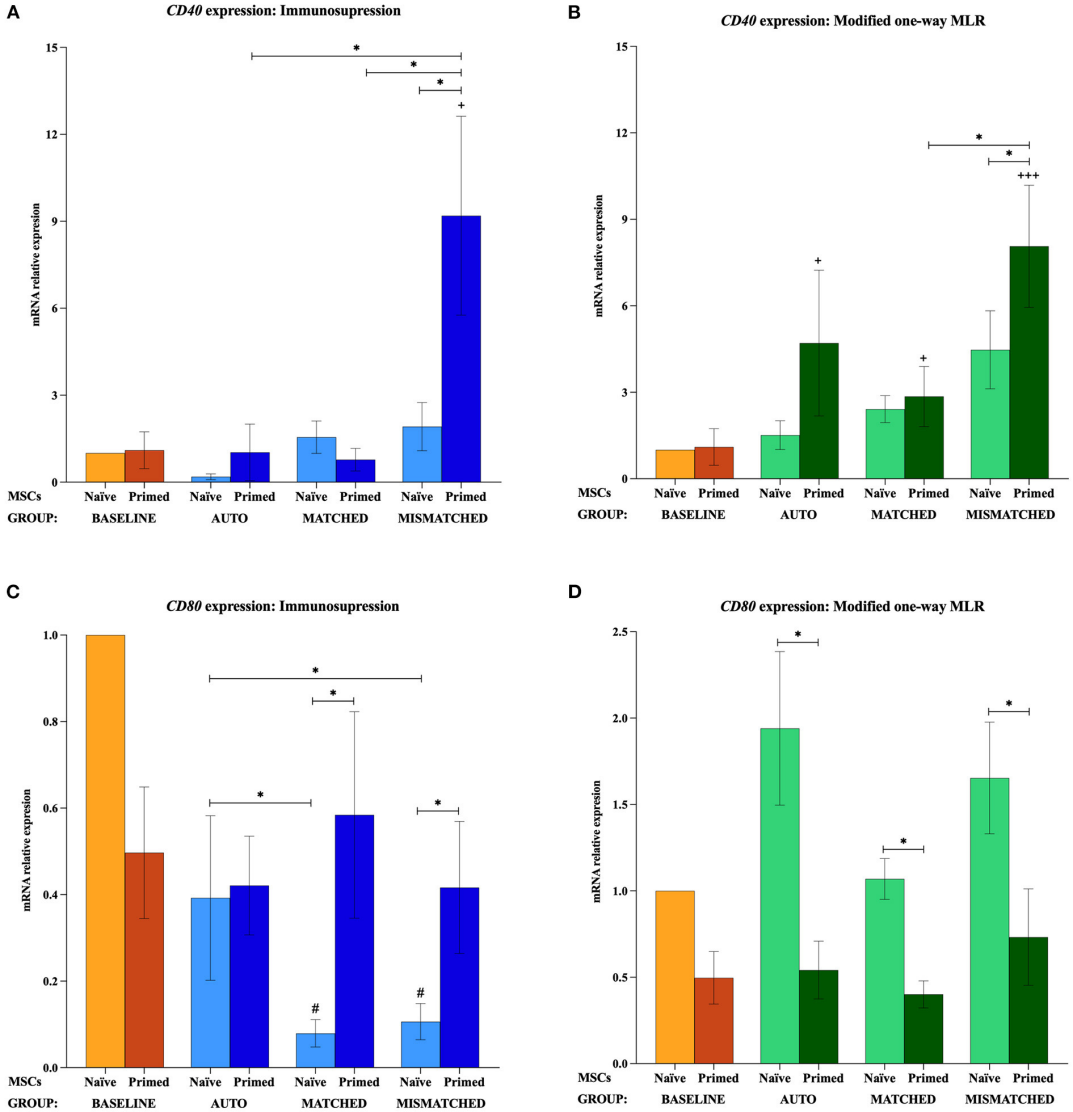
Changes in costimulatory molecules *CD40* and *CD80* expression by equine mesenchymal stem cells (MSCs) in the different scenarios. **(A)** *CD40* and **(C)** *CD80* mRNA relative expression before (baseline; orange bars) and after equine MSC-naive (light blue bars) and MSC-primed (dark blue bars) were exposed *in vitro* to phytohemagglutinin (PHA)-activated peripheral blood lymphocytes (PBLs) (immunosuppression assays). **(B)** *CD40* and **(D)** *CD80* mRNA relative expression before (baseline; orange bars) and after MSC-naive (light green bars) and MSC-primed (dark green bars) were exposed *in vitro* to resting PBLs [modified one-way mixed lymphocyte reaction (MLR) assays]. Co-cultures of MSCs and PBLs were autologous (*n* = 3) or allogeneic, matched (*n* = 8), or mismatched (*n* = 7) for the major histocompatibility complex. Changes in gene expression are represented as mean ± S.E.M of the relative mRNA expression, using baseline MSC-naive as reference sample (light orange bar, value 1). Significant differences in each condition compared with the baseline MSC-naive (light orange bar) are represented by hashes (#) above the corresponding bar (^#^*p* < 0.05). Significant differences compared with the baseline MSC-primed (dark orange bar) are represented by a cross (+) above the corresponding bar (^+^*p* < 0.05; ^+++^*p* < 0.001). Significant differences between experimental conditions are represented by a squared line with an asterisk (^*^p < 0.05).

The gene expression of *MHC-I, MHC-II, CD40*, and *CD80* was not increased by the priming at the baseline (MSCs cultured alone). *MHC-I* and *MHC-II* were only upregulated when MSCs were both primed and exposed to lymphocytes, regardless of these being activated or resting, autologous or allogeneic MHC-matched/-mismatched (Figure 5). Activated lymphocytes also induced *CD40* upregulation in MSC-primed but only if mismatched, while resting lymphocytes produced this increase in all the three types of co-cultures. On the contrary, activated lymphocytes tended to downregulate *CD80* expression in MSCs, and resting lymphocytes tended to increase it but only in MSC-naive (Figure 6).

#### Effect of activated lymphocytes on equine MSC immunogenic profile

When MSC-primed were exposed to activated matched or mismatched lymphocytes, *MHC-I* was significantly overexpressed compared with MSC-naive (*p* < 0.05 for both conditions; Figure 5A), and *MHC-II* increased significantly compared with the baseline (*p* < 0.05 for both conditions; Figure 5C). *MHC-II* overexpression in MSC-primed was also significant compared with MSC-naive in the MHC-matched co-cultures (*p* < 0.05). However, *CD40* was upregulated only in MSC-primed exposed to activated mismatched lymphocytes, and this increase was statistically significant compared with the baseline, the corresponding MSC-naive, and the MSC-primed exposed to autologous and matched activated lymphocytes (*p* < 0.05 for all conditions; Figure 6A). On the other hand, activated lymphocytes led to a reduction of *CD80* expression in MSC-naive in matched and mismatched co-cultures compared with the baseline, the corresponding MSC-primed, and the autologous co-culture (*p* < 0.05 for all conditions; Figure 6C).

#### Effect of resting lymphocytes on equine MSC immunogenic profile

The exposure to resting lymphocytes upregulated *MHC-I, MHC-II*, and *CD40* in MSC-primed compared with MSC-naive, while *CD80* was increased in MSC-naive compared with MSC-primed. Specifically, *MHC-I* increased in MSC-primed in both matched and mismatched co-cultures (*p* < 0.05 for both conditions), this upregulation being significantly higher in the mismatched co-culture than the matched (*p* < 0.05) and the baseline (*p* < 0.05; Figure 5B). In line with this, *MHC-II* and *CD40* increased compared with the baseline in MSC-primed exposed to all the three types of co-cultures (*MHC-II: p* < 0.001 autologous, *p* < 0.01 matched, *p* < 0.001 mismatched; *CD40: p* < 0.05 autologous, *p* < 0.05 matched, *p* < 0.001 mismatched) (Figures 5D, 6B). Moreover, *MHC-II* was significantly overexpressed compared with MSC-naive in the allogeneic matched and mismatched co-cultures (*p* < 0.05 matched, *p* < 0.01 mismatched). Similarly, the highest *CD40* expression was detected in MSC-primed exposed to mismatched resting lymphocytes, which was significantly increased compared with the corresponding MSC-naive (*p* < 0.05) and the matched co-culture (*p* < 0.05; Figure 6B). In contrast to these findings, the expression of *CD80* in MSCs exposed to resting lymphocytes was higher in MSC-naive than in MSC-primed in all the three co-cultures (*p* < 0.05 for all conditions; Figure 6D).

## Discussion

The role of the immunomodulation and immunogenicity of equine MSCs seems to be key for their therapeutic actions and to evade the immune system in the allogeneic administration. The environment that is encountered by MSCs greatly influences their immune properties, and the changes experienced by these cells in response can either benefit their potency (i.e., increased regulatory capacity) or compromise their effectiveness and safety (i.e., immune targeting and elimination) (6). Therefore, the knowledge on how MSCs respond to different stimuli is key to optimize cell therapies for veterinary and human patients. Although several studies have explored the effects of licensing MSCs with different cytokines and ligands, as well as the changes elicited by these MSCs on different populations of immune cells (9, 32), little is known about how immune cells lead to changes in equine MSCs that may have therapeutic implications. To the best of authors' knowledge, this is the first report on the changes experienced by equine MSCs in their immunomodulatory and immunogenic profiles upon exposure to cytokine priming and/or lymphocytes in different combinations (activated/resting; autologous/MHC-matched/MHC-mismatched). According to our initial hypothesis, co-culture of equine MSCs with activated lymphocytes resulted in an increased gene expression and secretion of immunomodulatory molecules, especially in MSC-primed. However, resting lymphocytes did not elicit remarkable changes, except when MSCs were previously primed and MHC-mismatched. As we hypothesized, a moderate activation of the equine MSC immunogenic profile was also observed, more markedly for MSC-primed but similar between activated and resting lymphocytes. We also hypothesized that MHC-mismatched MSCs would display similar immunomodulation but increased immunogenicity, which we indeed observed mostly for MSC-primed. Interestingly, the increased expression of immunogenic markers seemed to be accompanied by a further activation of the regulatory profile, which might equilibrate the balance between both properties in equine MSCs.

Prior to engaging into further discussion of our results, it is important to bear in mind the limitations of this study. First, the sample size in the experiments presented is limited due to the implications of working with a large species such as the horse (2), and it is particularly small for the baseline measurements and the autologous co-cultures (*n* = 3). Related to this, even though all horses enrolled in this study had similar characteristics (age, origin, breed, weight), the baseline gene expression of their MSCs and their response to the different conditions considerably varied. Thus, the inter-individual variability among the different donors can also be considered as a limitation and may have prevented to observe further significant differences. To account for this variability, the values of each donor were normalized compared with their corresponding baseline values. In addition, the difficulty in finding MHC-homozygote MSC donors that can be paired with PBL donors should be considered, taking the high variability of ELA haplotypes into account (10, 17). Another limitation is that not all of the gene expression results could be compared to the secretion of the molecule or its surface expression. There are some contradictory reports on iNOS2 and IDO1 activity in the equine MSC supernatant (26). For instance, Cassano et al. (33) described that the increased gene expression of *IDO1* by equine MSCs upon IFNγ stimulation is not enough to be translated into an actual increase in *IDO1* activity. Therefore, we decided to assess the gene expression of these enzymes and focus on IL6 and PGE_2_ to conduct ELISA as the role of these mediators has been more consistently reported in equine MSCs (34). The surface expression of the other molecules could not be assessed partly because of the lack of appropriate antibodies for the equine species (35) and partly because of the insufficient number of MSCs to conduct both RT-qPCR and flow cytometry. Therefore, gene expression was prioritized in this study as it has been widely used in the equine MSC literature and can provide relevant information on the changes experienced by these cells (7, 36).

Several authors agree that PGE_2_ is the primary mediator responsible for inhibition of lymphocyte proliferation by equine MSCs from different sources, including BM, adipose tissue (AT), umbilical cord blood (CB), and umbilical cord tissue (CT) (4, 33). PGE_2_ is produced by COX2, so both the concentration of the soluble molecule and the gene expression of the enzyme are usually assessed in equine studies (26, 27, 30). The secretion of IL6 may not be mainly involved in the inhibition of T-cell proliferation by equine MSCs (30), but studies in other species agree that IL6 may contribute to a more efficient immunosuppression of B lymphocytes (37, 38). iNOS2 is mainly involved in the immune regulatory effects of rodent MSCs, while MSCs from other mammalian species (e.g., monkey, pig, dog, cattle, and human) preferentially use IDO1 (38–41). Equine studies have reported different results regarding iNOS2 participation. Carrade et al. (4) described that NO production by equine MSCs varies from different tissue sources, and subsequent studies have shown that iNOS2 inhibition does not change the inhibitory effect of equine MSCs on lymphocyte proliferation (26, 30, 42). IDO1 activity does not seem to be involved in the capacity of equine MSCs to inhibit allogeneic lymphocyte proliferation (26) but may participate in maintaining this suppressive effect (42). In addition to soluble mediators, cell–cell interactions between MSCs and lymphocytes *via* adhesion molecules may increase the effectiveness of the MSC immunomodulation (43). Indeed, some authors agree that *VCAM1* is only expressed upon direct close contact between MSCs and lymphocytes, so this molecule may play a key role in the immunosuppressive functions of MSCs (37).

The immunomodulatory actions of MSCs are closely related not only with their therapeutic mechanisms but also with their ability to evade the immune system, in which the expression of immunogenic molecules also plays a role. Expression of MHC-I by MSCs may result in their immune recognition and elimination since cytotoxic T cells attack foreign cells bearing MHC-I receptors that are bound to an alloantigen (44). On the other hand, natural killer (NK) cells can attack cells lacking MHC-I on their surface (45), so the expression of MHC-I, although weak, protects MSCs from NK cell-mediated elimination (46). In addition, expression of MHC-II can also lead to MSC targeting, so its lack confers these cells the ability to escape immune recognition by CD4 helper cells (46). The cell surface expression of MHC-I and II on equine MSCs vary from one donor to another and even among MSC samples (47, 48), so a recent study proposed to classify equine MSCs as MHC class II-high or -low (7). According to this, MSCs from the three donors in this study would be classified as MHC-II low but would be considered MHC-II high after cytokine priming (16). In addition to MHC complexes, other costimulatory molecules are involved in the antigenic presentation and may have an impact on the immune recognition of MSCs. CD40 plays an important role in allograft rejection (49, 50), and its expression in human MSCs may contribute to an effective activation of T cells (51). Furthermore, the coupling ligand of CD80 to the CD28 receptor is the first signaling pathway necessary for T-cell costimulation and enhances T-cell proliferation and cytokine secretion (51). Equine MSCs from peripheral blood and CB-MSCs showed a moderate to strong expression of these costimulatory molecules (35); however, their role in the immune properties of equine MSCs is largely unknown.

All the molecules and/or genes assessed in the current study have been reported to change its expression and/or secretion in response to an inflammatory environment. Specifically, TNFα and IFNγ are considered inductors of immunomodulatory mediators by MSCs from different species and sources, including the horse (38, 42, 50, 52). In this study, the gene expression and secretion of immunomodulatory molecules were induced after the priming in MSCs, whereas the immunogenic markers were not upregulated in this condition; that is, the cytokine exposure alone could activate the immune regulatory profile of equine BM-MSCs without affecting their immunogenic profile. Similarly to our findings, TNFα and IFNγ have been reported to upregulate *VCAM1* in murine MSCs, and this priming rendered MSCs more adhesive to CD8+ T cells, CD4+ T cells, and CD3+ T cells (53). Other reports also observed an upregulation of the expression and secretion of *IL6* after cytokine priming of equine MSCs (15, 42, 54), as well as of the gene expression of *COX2* and secretion of PGE_2_ after exposure to TNFα and IFNγ (5, 36, 42). Priming with IFNγ also resulted in significant induction of *iNOS2* and *IDO1* expression in equine MSCs in previous works (36, 42), whereas other studies did not detect its expression after priming (33). Interestingly, MSCs from the three donors in this study showed no expression of *iNOS2* at the baseline unless MSCs were primed, suggesting that some regulatory factors are only expressed upon activation, and this licensing may depend on the type and degree of stimulation.

Even though there are several reports on the effect of cytokine priming on equine MSCs, there is limited information on the changes in the immune profile of these cells after being challenged by lymphocytes. This would more closely resemble the *in vivo* environment that MSCs encounter after administration, in which the immune system may already be activated (inflammation at the injury site, immune-mediated disease) or may be stimulated in response to the MSCs (immunogenic recognition). In our study, when MSC-naive were exposed to activated lymphocytes, the gene expression of *IL6, COX2*, and *iNOS2* was upregulated, and the secretion of IL6 and PGE_2_ increased to levels similar to or even higher than those after cytokine priming alone. Moreover, in MSC-primed, the baseline expression and secretion of the same mediators were further increased after the co-culture with activated lymphocytes. The consensus on the role of PGE_2_ and IL6 in equine MSC immunomodulation is quite broad, so their upregulation upon cytokine priming and/or exposure to activated lymphocytes observed in this study agrees with previous knowledge (30, 36). However, as aforementioned, there is controversy on the participation of *IDO1* and *iNOS2* in the regulatory mechanisms of equine MSCs. Whereas, the dynamics of *iNOS2* gene expression followed the same trend as *IL6* and *COX2* in this study, the expression of *IDO1* decreased in MSC-primed co-cultured with activated lymphocytes. Some studies did not detect IDO1 activity in the supernatants of co-cultures with equine MSCs and PBMCs, either PHA activated or not, suggesting that this pathway may not be functionally active and that equine MSCs failed to produce IDO1 in the presence of stimulated T cells (4, 26). Along with our findings, it may be suggested that the induction of this molecule may depend on the type of stimuli and may require further stimulation to remain active. Similarly to that observed for *IDO1, VCAM1* was also downregulated in MSC-primed exposed to activated lymphocytes, which seemed to be influenced by the type of co-culture. While we do not have a clear hypothesis for this observation, it may also be related with a potentially different regulatory ability of equine MSCs in different contexts. Overall, these findings show that activated lymphocytes constitute an environment able to stimulate equine MSCs, so these could be similarly licensed *in vivo*. Furthermore, if MSCs are already primed by cytokines, activated lymphocytes can further contribute to the upregulation of modulatory factors, suggesting that the effect of both stimuli might be additive.

On the contrary, when MSCs were exposed to resting lymphocytes, IL6 secretion and the majority of the immunomodulatory genes were downregulated in both MSC-naive and MSC-primed. *COX2* gene expression followed the same pattern of downregulation, but PGE_2_ secretion differed from this tendency. Even though the deviation observed in the PGE_2_ concentration prevents outlining a clear trend, higher levels of PGE_2_ were overall observed in MSCs exposed to resting lymphocytes than in MSCs alone. The regulation on eicosanoid pathways is complex and happens at different levels (55), and the data of this study do not allow establishing a definitive explanation for this discrepancy between gene expression and molecule secretion. We have two potential hypotheses for this observation: First, an initial activation of *COX2* could have happened followed by a downregulation, but PGE_2_ secreted upon activation could still be present in the supernatant. Second, a regulatory loop could have taken place in which PGE_2_ in the medium would have downregulated the expression of *COX2*. In spite of this discrepancy, the general pattern was toward downregulation of the equine MSC modulatory profile, which may be due to the lack of further activation exerted by resting lymphocytes. Thus, the baseline expression of regulatory markers will be reduced after 5 days if MSCs are no longer stimulated. Similarly, a previous report from our group found that the overexpression of these markers induced by cytokine priming diminishes after 7 days in MSCs cultured alone (15). Therefore, it can be hypothesized that resting lymphocytes did not promote or maintain the licensing of equine MSCs, indirectly reflecting their own lack of activation.

Interestingly, the higher regulatory profile in MSC-primed exposed to resting MHC-mismatched lymphocytes may be related to the activation of the latter upon encountering mismatched MSCs as these also presented increased expression of *MHC I, MHC-II*, and *CD40* in this condition. The overexpression of these immunogenic markers may facilitate the allo-recognition of MSCs by lymphocytes, which response might activate the regulatory profile of MSCs, and this could facilitate their immune escape. The increased expression of *MHC-I* and *MHC-II* after priming equine MSCs has been previously reported to different extent (9, 15, 36). Similarly, previous studies reported an increase in *MHC-II* expression after equine MSCs were exposed to conditioned media from PBMCs (26). The overexpression of *MHC-I* and *MHC-II* in MSC-primed alone was not observed in this study, but these markers were induced after exposure of MSC-primed to both activated and resting lymphocytes, regardless of the MHC compatibility. It has also been reported that the costimulatory molecule *CD40* could be upregulated on MSCs under inflammatory conditions in different species. In human studies, ~50% of AT-MSCs expressed *CD40* (50), and cytokine priming enhanced the inhibitory function of MSCs derived from tonsils when expression of *IDO1* and *CD40* increased (49). However, in the conditions of this study, *CD40* was not modified by priming by exposure to activated lymphocytes, separately, and *CD40* was only overexpressed upon simultaneous priming and co-culture with mismatched activated lymphocytes. Previous studies reported that cytokine priming of human MSCs did not increase *CD80* expression as it happens with other immune markers; furthermore, *CD80* expression could be downregulated in MSCs after priming (56, 57). Similarly, in this study, *CD80* expression was reduced upon priming, and its expression after co-culture with activated lymphocytes remained low. Curiously, *CD80* expression was higher in MSC-naive after exposure to resting lymphocytes, differing from the tendencies observed for other genes. Taken together, these findings suggest that different simultaneous stimuli are needed to induce the immunogenic profile of equine MSCs, while a single stimulus would be able to induce their immune regulatory potential.

Even though these observations were not directly correlated with functional implications in this work, it is worth discussing how these changes might translate into immune suppression and immune recognition mechanisms. Previous studies have found that the ability of pro-inflammatory primed equine MSCs to suppress the proliferation of allogeneic activated T cells is enhanced, but these primed cells could also lead more easily to immune activation (32, 42), which would agree with the expression patterns seen in our study. Furthermore, a previous work from our group found that equine MSCs, either naive or primed, were able to change the frequency and proliferation of different subsets of equine activated or resting lymphocytes (16).

In the immunosuppressive assays (activated PBLs) of the current study, the increase in regulatory gene expression and secretion by MSC-primed would agree with our previous results in which the capacity of suppressing CD3+ T cells, CD4+ T cells, CD8+ T cells, and B cells was enhanced in MSC-primed compared with MSC-naive (16). Specifically, PGE_2_ can downregulate the proliferation of cytotoxic T cells and B-cell activation (58) and is considered the main factor conferring the ability of equine MSCs to suppress lymphocyte proliferation (26, 30, 42). Even though the role of IL6 in equine MSC immunomodulation needs further elucidation, it has been seen in other species that this molecule is involved in the suppression of B lymphocytes (37, 38). Therefore, the higher *IL6* expression and secretion by MSC-primed might be related with the stronger suppression of B cells in immunosuppression assays and the lack of induction of B cells in modified one-way MLR assays, as observed in a previous study (16). Furthermore, the changes in the profile of equine MSCs may also be implicated in their ability to induce changes in the subpopulation of T reg cells. Human BM-MSCs are known to promote immune suppression by inducing the production of T reg, which would downregulate the proliferation of CD8+ cytotoxic T cells (59). Specifically, PGE_2_ secreted by human MSCs induces CD4+ T-cell differentiation into Tregs (39), and it has also been observed in other species that the overexpression of *COX2* prevents the downregulation in the number of CD4+CD25+ Treg cells (58). While this is not well established in horses, in our previous work, the presence of equine MSC-naive and MSC-primed increased the percentage of CD4+ CD25^high^ T cells in a population of activated lymphocytes (16), which might be related with the overexpression of *COX2* and increased secretion of PGE_2_ observed in the present work. Even though a direct relation cannot be established as these are found in separate studies, it is worth mentioning that the conditions in which equine MSCs showed higher *COX2* expression and PGE_2_ secretion in the current study were comparable to those that displayed the highest suppressive capacity for CD8+ T cells (16).

By conducting modified one-way MLR assays with resting lymphocytes, we previously described that MSC-primed induced a proliferative response in cytotoxic and helper T cells, and this immunogenic response was more marked when the lymphocytes were MHC-mismatched with the MSCs. Similarly, MHC-mismatched MSC-primed can induce the proliferation of resting CD8+ cytotoxic cells, suggesting their immune recognition, but this condition also activates the T reg cells, which may counter the activation of the first ones (16). Interestingly, in the present study, we observed an induction of the regulatory profile precisely in MSC-primed exposed to resting mismatched lymphocytes, which may be a licensing effect conducted by the activation of the immune cells.

Regarding changes linked to the different MHC matching between equine MSCs and lymphocytes, we overall observed higher expression and/or secretion of regulatory molecules in MSCs in the autologous co-cultures with activated lymphocytes, followed by allogeneic MHC-matched and lastly by MHC-mismatched co-cultures. This observation might relate with a trend previously found for autologous MSCs to further elicit immune suppression of PHA-stimulated PBLs, followed by MHC-matched and mismatched MSCs (16). These are general patterns that cannot be directly compared, and this tendency is not reflected as significant differences among co-cultures for all the mediators assessed; however, it is particularly well-represented by *VCAM1*. Actually, it has been reported that the higher the expression level of *VCAM1*, the greater the MSC inhibitory capacity (53).

The results of this study show that an inflammatory environment can induce a regulatory profile in equine MSCs but can also increase their immunogenic expression. Similar findings have been previously reported, but the novelty of this study is to shed light on the effect of different conditions by directly comparing several scenarios. First, both cytokine priming and activated lymphocytes are able to induce the regulatory profile of equine MSCs separately, but the changes experienced by equine MSCs are different. Furthermore, the action of both stimuli appears to be additive, especially for immunogenic markers. Second, when MSCs have been primed and are specifically exposed to MHC-mismatched lymphocytes, their regulatory profile is further increased. This has been particularly noted when the co-culture was caried out with resting lymphocytes, where MSC-primed also increased their expression of the immunogenic markers *MHC-I, MHC-II*, and *CD40*. We hypothesize that such upregulation may facilitate the allo-recognition of foreign MSCs, and thus, the activation of lymphocytes could induce the regulatory profile of MSCs, which, at the same time, would facilitate their immune escape. This potential explanation is in line with the concept of immunomodulation–immunogenicity balance (6, 14), according to which MSCs are able to evade the immune response by equilibrating their capacities to suppress and to activate it. In conclusion, these findings highlight the plasticity of MSCs to respond to stimuli of different nature and degree, and the key role of the balance between their immune regulatory and immunogenic properties. This study also underscores the complexity of the interactions between MSCs and the immune system, giving clues on how these cells may behave once they are administered in the patients, which also can shed light on the mixed results usually obtained in *in vivo* studies. Although the actual clinical impact of these findings remains to be further explored, this information can facilitate the development of *in vivo* studies to further understand the immune properties of equine MSCs, which are key in the path toward safer and more effective cell therapies.

## Data availability statement

The original contributions presented in the study are included in the article/Supplementary material, further inquiries can be directed to the corresponding author/s.

## Ethics statement

The animal study was reviewed and approved by Advisory Ethics Committee for Animal Research from the University of Zaragoza (Project License PI 15/16).

## Author contributions

LB, AC, EB, and IG were responsible for acquisition of data. AC, LB, IG, FV, and CR were responsible for analysis and interpretation of data. AR, AV, EB, MG-M, and FV provided technical support. AR, FV, and CR provided important conceptual guidance. CR was responsible for obtaining funding. LB and AC drafted the manuscript. FV, AR, AV, EB, MG-M, IG, and CR revised the manuscript for important intellectual content. All authors substantially contributed to the conception, design of the study, approved the final submitted version of the manuscript, and agree to be accountable for all aspects of the work in ensuring that accuracy or integrity of any part of the work are appropriately investigated and resolved. All authors have read and agreed to the published version of the manuscript.

## Funding

This research was mainly funded by Ministerio de Industria, Economia y Competitividad, Spain (Grant No. AGL2017-84411-P), Ministerio de Ciencia e Innovación, Spain (Grant No. PID2020-116352GB-100), and partially financed by the Gobierno de Aragón (Grupo de Investigación A19_17R, LAGENBIO). AC was supported by a Ph.D. fellowship from the Gobierno de Aragón and co-funded by the European Social Fund.

## Conflict of interest

The authors declare that the research was conducted in the absence of any commercial or financial relationships that could be construed as a potential conflict of interest.

## Publisher's note

All claims expressed in this article are solely those of the authors and do not necessarily represent those of their affiliated organizations, or those of the publisher, the editors and the reviewers. Any product that may be evaluated in this article, or claim that may be made by its manufacturer, is not guaranteed or endorsed by the publisher.

## Abbreviations

AT: adipose tissue
B2M: beta-2-microglobulin
BM: bone marrow
CB: umbilical cord blood
CT: umbilical cord tissue
COX2: cyclooxygenase 2
DMEM: Dulbecco's modified Eagle medium
DMSO: dimethyl sulfoxide
ELA: equine leukocyte antigen
FBS: fetal bovine serum
GAPDH: glyceraldehyde 3-phosphate dehydrogenase
IDO1: indoleamine 2,3-dioxygenase 1
IFNγ: interferon gamma
IL6: interleukin 6
iNOS1: inducible nitric oxide synthase 1
iNOS2: inducible nitric oxide synthase 2
iPSCs: induced pluripotent stem cells
MHC: major histocompatibility complex
MLR: mixed lymphocyte reaction
MSC: mesenchymal stem cell
NK: natural killer
PBL: peripheral blood lymphocyte
PBS: phosphate-buffered saline
PGE_2_: prostaglandin E2
PHA: phytohemagglutinin
RT-qPCR: real-time quantitative polymerase chain reaction
TNFα: tumor necrosis factor alpha
VCAM1: vascular cell adhesion molecule 1.
