# Supplementary material for: The immunomodulation–immunogenicity balance of equine Mesenchymal Stem Cells (MSCs) is differentially affected by the immune cell response depending on inflammatory licensing and major histocompatibility complex (MHC) compatibility
Source: Front Vet Sci. 2022 Oct 20;9:957153. doi: 10.3389/fvets.2022.957153 (PMC9632425; doi:10.3389/fvets.2022.957153)
Supplement: Supplementary file 1 [file Table_1.DOCX]

Supplementary Material

**Table S1.** List of major histocompatibility complex (MHC) microsatellite haplotypes identified in the horses enrolled in the study.

|  | MHC class I | | MHC class III | | MHC class II | | | | | |  | |
| --- | --- | --- | --- | --- | --- | --- | --- | --- | --- | --- | --- | --- |
| Microsatellite  loci | UMNJH-38 | COR110 | ABGe9019 | UMNe65 | ABGe9030 | EQMHC 1 | COR112 | COR113 | UM011 | COR114 |  |  |
| Horses ID |  |  |  |  |  |  |  |  |  |  | **Haplotype** |  |
| **D1** | 165 | 221 | 301 | 261 | 215 | 190 | 262 | 270 | 179 | 241 | HapPRE10 |  |
|  | 165 | 221 | 301 | 261 | 215 | 190 | 262 | 270 | 179 | 241 | HapPRE10 |  |
| R1 | 165 | 221 | 301 | 261 | 215 | 190 | 262 | 270 | 179 | 241 | HapPRE10 |  |
|  | 156 | 215 | 301 | 261 | 215 | 190 | 262 | 270 | 179 | 241 | HapPRE10-like |  |
| R2 | 165 | 221 | 301 | 261 | 215 | 190 | 262 | 270 | 179 | 241 | HapPRE10 |  |
|  | 156 | 221 | 320 | 250 | 219 | 190 | 254 | 270 | 172 | 249 | HapMAI06 |  |
| R3 | 165 | 221 | 301 | 261 | 215 | 190 | 262 | 270 | 179 | 241 | HapPRE10 |  |
|  | 156 | 207 | 318 | 263 | 215 | 184 | 262 | 260 | 172 | 243 | HapPRE31 |  |
| **D2** | 156 | 217 | 312 | 261 | 205 | 194 | 258 | 260 | 169 | 243 | HapMAI04 |  |
|  | 156 | 217 | 312 | 261 | 205 | 194 | 258 | 260 | 169 | 243 | HapMAI04 |  |
| A1 | 156 | 217 | 312 | 261 | 205 | 194 | 258 | 260 | 169 | 243 | HapMAI04 |  |
|  | 156 | 205 | 305 | 253 | 205 | 194 | 266 | 268 | 174 | 234 | HapPRE01 |  |
| A2 | 156 | 217 | 312 | 261 | 205 | 194 | 258 | 260 | 169 | 243 | HapMAI04 |  |
|  | 156 | 207 | 312 | 263 | 211 | 192 | 264 | 270 | 172 | 249 | Unknown1 |  |
| **D3** | 156 | 221 | 314 | 259 | 215 | 190 | 262 | 272 | 169 | 255 | HapPRE11 |  |
|  | 156 | 221 | 314 | 259 | 215 | 190 | 262 | 272 | 169 | 255 | HapPRE11 |  |
| C1 | 156 | 221 | 314 | 259 | 215 | 190 | 262 | 272 | 169 | 255 | HapPRE11 |  |
|  | 156 | 221 | 312 | 261 | 205 | 190 | 262 | 270 | 180 | 245 | Unknown2 |  |
| C2 | 156 | 221 | 314 | 259 | 215 | 190 | 262 | 272 | 169 | 255 | HapPRE11 |  |
|  | 156 | 211 | 301 | 259 | 209 | 192 | 262 | 268 | 174 | 234 | A2* |  |
| C3 | 156 | 221 | 314 | 259 | 215 | 190 | 262 | 272 | 169 | 255 | HapPRE11 |  |
|  | 156 | 207 | 314 | 261 | 215 | 190 | 262 | 270 | 180 | 247 | HapPRE26 |  |

MHC homozygous horses are indicated in bold. Asterisks indicate haplotypes that have been previously identified in other horse breeds (17).
