# Supplementary material for: The immunomodulation–immunogenicity balance of equine Mesenchymal Stem Cells (MSCs) is differentially affected by the immune cell response depending on inflammatory licensing and major histocompatibility complex (MHC) compatibility
Source: Front Vet Sci. 2022 Oct 20;9:957153. doi: 10.3389/fvets.2022.957153 (PMC9632425; doi:10.3389/fvets.2022.957153)
Supplement: Supplementary file 2 [file Data_Sheet_1.DOCX]

Supplementary Material


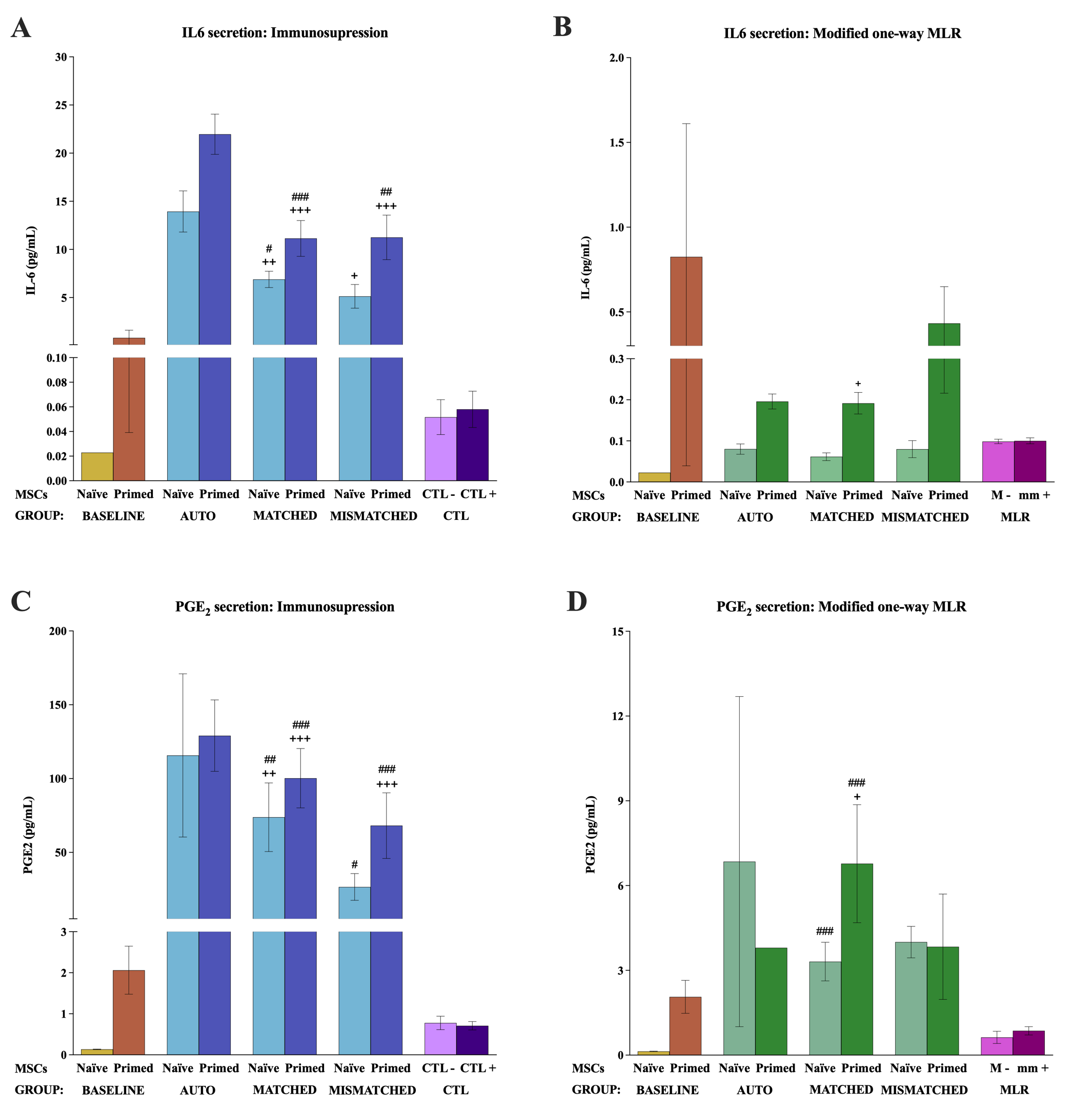


**Figure S1.** Significant differences over controls are presented for interleukin 6 (IL6) and prostaglandin E2 (PGE_2_) secretion. The graphs in this figure are modifications of those presented in Figure 2. C and D (IL6) and Figure 3. C and D (PGE_2_) to include the concentration of the mediators found in the supernatant of the controls, and to show the significant differences of the experimental conditions over these controls. These results are not presented in the main figures as the mediators’ secretion by lymphocyte controls is not relevant for the study and was just assessed to corroborate that the mesenchymal stem cells (MSCs) were the main contributors to this secretion. We considered that presenting multiple comparison in the same figure would prevent highlighting the most relevant findings for the study, so the comparison over controls is separately presented in this supplementary material. Data are presented as Mean ± S.E.M of the concentration (pg/mL) of IL6 (**A***,* **B**) and PGE_2_ (**C**, **D**) found in the supernatants of equine MSCs unstimulated (MSC-naïve) or cytokine primed (MSC-primed) before (baseline) and after their co-culture with autologous (*n* = 3) or allogeneic matched (*n* = 8) or mismatched (*n* = 7) lymphocytes, either activated (**A**, **C**; immunosuppression assays) or resting (**B**, **D**; modified one-way mixed lymphocyte reactions, MLR). In immunosuppression assays, lymphocytes from all donors were cultured alone as positive (phytohemagglutin isoform P activation; CTL+, dark purple bar) and negative (unstimulated; CTL-, light purple bar) controls. In modified one-way MLRs, positive and negative controls were set by establishing, respectively, matched (MLR M-, light pink bar) and mismatched (MLR mm+, dark pink bar) classic MLRs using responder PBLs from each donor. Significant differences over the positive controls are presented by ‘+’ (+ = *p* < 0.05; ++ = *p* < 0.01; +++ = *p* < 0.001) and over the negative controls as ‘#’ (# = *p* < 0.05; ## = *p* < 0.01; ### = *p* < 0.001) placed above the corresponding bars of experimental conditions.
